# Loss of Quaking RNA binding protein disrupts the expression of genes associated with astrocyte maturation in mouse brain

**DOI:** 10.1038/s41467-021-21703-5

**Published:** 2021-03-09

**Authors:** Kristina Sakers, Yating Liu, Lorida Llaci, Scott M. Lee, Michael J. Vasek, Michael A. Rieger, Sean Brophy, Eric Tycksen, Renate Lewis, Susan E. Maloney, Joseph D. Dougherty

**Affiliations:** 1 Department of Genetics, Washington University School of Medicine, Saint Louis, MO 63110 USA; 2 Department of Psychiatry, Washington University School of Medicine, Saint Louis, MO USA; 3 Division of Biomedical and Biological Sciences, Washington University School of Medicine, Saint Louis, MO USA; 4 Genome Technology Access Center, McDonnell Genome Institute, Washington University School of Medicine, Saint Louis, MO USA; 5 Hope Center, Washington University School of Medicine and Saint Louis University, Saint Louis, MO USA

**Keywords:** Transcriptomics, Astrocyte

## Abstract

Quaking RNA binding protein (QKI) is essential for oligodendrocyte development as myelination requires myelin basic protein mRNA regulation and localization by the cytoplasmic isoforms (e.g., QKI-6). QKI-6 is also highly expressed in astrocytes, which were recently demonstrated to have regulated mRNA localization. Here, we define the targets of QKI in the mouse brain via CLIPseq and we show that QKI-6 binds 3′UTRs of a subset of astrocytic mRNAs. Binding is also enriched near stop codons, mediated partially by QKI-binding motifs (QBMs), yet spreads to adjacent sequences. Using a viral approach for mosaic, astrocyte-specific gene mutation with simultaneous translating RNA sequencing (CRISPR-TRAPseq), we profile ribosome associated mRNA from QKI-null astrocytes in the mouse brain. This demonstrates a role for QKI in stabilizing CLIP-defined direct targets in astrocytes in vivo and further shows that QKI mutation disrupts the transcriptional changes for a discrete subset of genes associated with astrocyte maturation.

## Abstract (web summary)

Quaking RNA binding protein (QKI) is known for its role in oligodendrocyte maturation. Here, the authors define the QKI targets in the mouse brain and show that loss of QKI disrupts the expression of cell maturation-associated genes in astrocytes in vivo.

## Introduction

Post-transcriptional regulation of mRNA by RNA binding proteins (RBPs) is pervasive in the Central Nervous System (CNS). RBPs serve as the *trans*-acting factors that recognize *cis*-acting elements that are commonly found in the 3′UTR of localized transcripts^1^. These proteins may directly transport RNAs to distal processes^2^, alter transcript stability, or control translation^3,4^ to prevent ectopic expression during mRNA transport or to activate translation in response to a molecular cue, for example. While these processes have been widely studied in other cell-types, it has recently become apparent that astrocytes also must have substantial cytoplasmic post-transcriptional regulation: for example, a set of mRNAs enriched on ribosomes in peripheral astrocyte processes (PAPs) have been defined, consistent with sequence-regulated local translation^5^. Likewise, there are clear examples of post-transcriptional regulation, via expression of specific cytoplasmic RBPs, being an essential aspect of differentiation and maturation of neurons^6^, microglia^7^, and oligodendrocytes^8^. However, aside from elegant work on the Fragile-X Mental Retardation Protein (FMRP)^9,10^, post-transcriptional regulators of such processes in astrocytes largely remain unknown.

Quaking RNA binding protein (QKI) is the mammalian homolog of *C. elegans* GLD-1 and *D. melanogaster* HOW, and part of the signal transduction and activation of RNA (STAR) family of post-transcriptional RBPs. Members of this evolutionarily conserved family of proteins are characterized by conserved domains which are necessary for homodimerization and RNA binding^11^. STAR family proteins are important early in development as their expression regulates smooth muscle development, cell-fate, and germ-line development^12,13^. However, QKI expression in mice remains high in the brain throughout the lifespan^14,15^, suggesting ongoing regulatory roles in the CNS. QKI is predominantly expressed in glia in the brain^16^ and its function in oligodendrocytes has been well-studied: QKI plays a role in oligodendrocyte differentiation and development by regulating the stability of mRNAs that inhibit cell cycle progression and promote differentiation^17–19^. Further, cytoplasmic isoforms of QKI control the nuclear export, stability, and localization of myelin basic protein (MBP) mRNA to oligodendrocyte processes^8,20^.

Although most of our knowledge of QKI’s function in vivo comes from studies focused on oligodendrocytes, this protein is also expressed in astrocytes in both mice and humans^21,22^. In an astroglioma cell line, it has been shown that one QKI isoform stabilizes transcripts, particularly those induced by interferons^23^. Moreover, examination of PAP-localized transcripts from maturing astrocytes revealed a preponderance of QKI-binding motifs (QBMs) in their UTRs^5^. However, we still do not understand to which cytoplasmic targets QKI binds in vivo nor what the downstream effects on these targets are in the maturing postnatal brain. Furthermore, the study of this gene is complicated as full knockouts are embryonic lethal^12^, making it challenging to disentangle direct cellular function of QKI from indirect effects of disrupted development.

Here, we focus on the cytoplasmic isoform QKI-6 to comprehensively define its targets in the maturing brain. We test the hypothesis that QKI-6 disproportionately binds PAP-localized transcripts and define the consequences of QKI mutation on mRNAs in astrocytes in vivo. We find, by Cross-Linking and Immunoprecipitation (CLIP)-seq, many QKI-6 targets are indeed astrocytic and overlap with a subset of the transcripts enriched on PAP ribosomes. Further, we apply an approach using CRISPR technology coupled to cell-type-specific profiling to define roles for this gene on mRNAs in astrocytes in vivo. Finally, we validate a role for QKI in transcript stability for the CLIP-defined targets.

## Results

### QKI-6 binds translationally regulated mRNAs in astrocytes

**Fig. 1:**
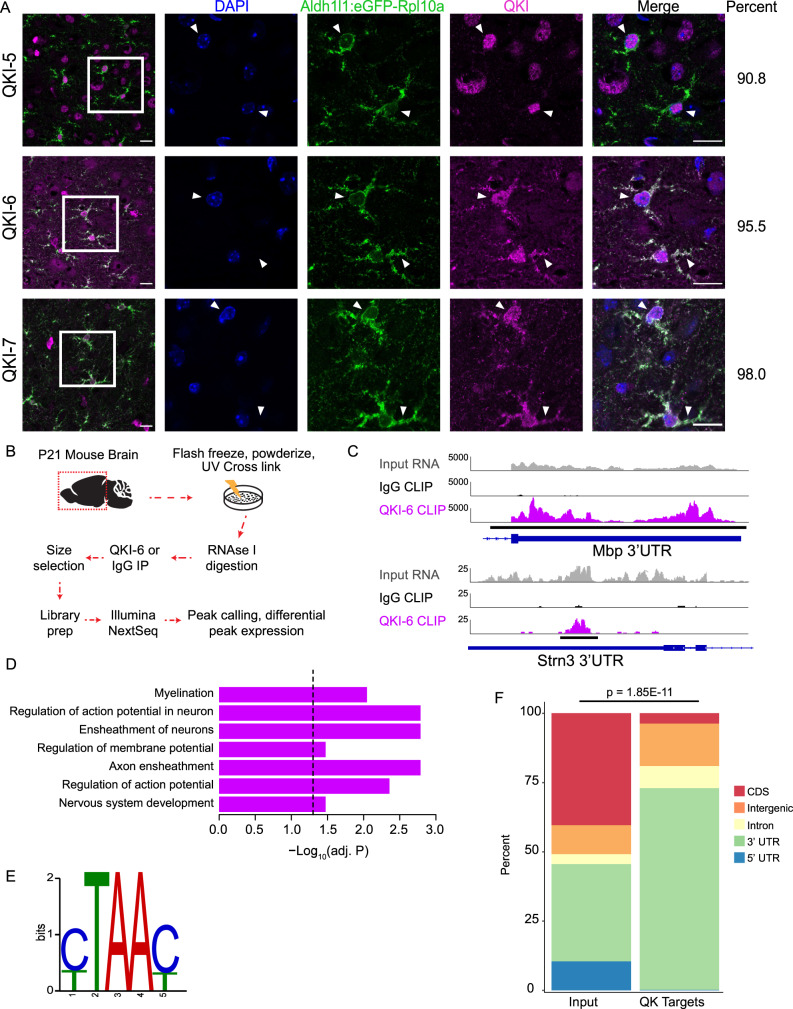
QKI-6 CLIP defines binding targets in developing forebrain in vivo. **A** Representative IF images reveal all QKI isoforms (magenta) are present in cortical astrocytes, in vivo. Left merged image = 40×, right 4 boxes are the boxed region magnified from the 40× image. Scale bar = 15 μm, right side: % of astrocytes showing strong QKI positivity for QKI-5 (208/229 cells), QKI-6 (213/223), and QKI-7 (199/203). Quantification was performed across at least 2 brains per QKI antibody. Source data are provided as a Source Data file. **B** QKI CLIP work flow. Brain icon is used under Creative Commons Attribution 4.0 International License (https://creativecommons.org/licenses/by/4.0/). **C** Browser shots of Input RNA-seq, QKI-6 IP vs IgG control reads along *Mbp* 3’UTR (top), as a control, and *Strn3* 3’UTR (bottom), an identified QKI CLIP target. Black lines under CLIP track indicate a called peak. Contiguous peaks are merged (e.g., for MBP). **D** Gene Ontology results for all significant biological processes across QKI-6 binding targets. Dotted line represents *p* = 0.05 from hypergeometric test with Benjamini–Hochberg FDR correction. **E** Top identified motif under QKI-6 peaks matches QBM. **F** Distribution of genome features for significant peaks in QKI-6 IP reveals enrichment of 3’UTRs. *P* value represents significance from two-sided Fisher’s exact test.

To understand how QKI regulation of mRNAs might affect astrocyte development and/or function, we first sought to determine which mRNAs were targeted by QKI in vivo. Although a few targets of QKI have been well studied in oligodendrocytes^18,20,24^, and 6–10 mRNAs have been shown to be altered in cultured astrocytes and related tumors after siRNA knockdown^25,26^, a compendium of all in vivo postnatal QKI targets has yet to be compiled. QKI has three major isoforms in humans and in mice, designated by their unique C-termini^27^, all of which are present in astrocytes^16^ as shown by colocalization with RPL10a-eGFP in Aldh1L1 transgenic bacTRAP mice (Aldh1L1:eGFP+; Fig. 1A). We chose to focus on QKI-6 for CLIP experiments for two reasons: first, QKI-5 is predominantly nuclear but QKI-6 and QKI-7 are both nuclear and cytoplasmic, and we posited that cytoplasmic isoforms are more likely candidates for translation regulation. Second, QKI-6 is expressed earlier in development than QKI-7^28^ suggesting an importance in early neuronal circuitry formation, a function largely governed by astrocytes. In addition, QKI-7 signal by immunofluorescence was weaker than QKI-6 (Fig. 1A). We found by immunofluorescence that >95% of Aldh1L1:eGFP+ astrocytes showed immunoreactivity for QKI-6 (Fig. 1A), thus supporting our rationale for studying QKI-6 in postnatal cortical astrocytes.

To define QKI-6 targets in all cells of maturing mouse forebrains, three independent biological replicates were homogenized and UV irradiated to cross-link (CL) protein-RNA complexes (Fig. 1B). We chose P21 because we previously found at this age that mRNAs enriched on PAP ribosomes contain more QBMs than expected by chance^29^. Subsequent QKI-6 immunoprecipitation (IP) or IgG IP (background control) were performed on CL lysates. QKI-6 IP clearly yielded more end-labeled RNA than controls, and a shift in QKI-6/mRNA complexes was detected with decreasing RNAse concentrations, as expected (Supplementary Fig. 1). After generation of CLIPseq libraries from QKI-6 CLIP, IgG IP (to control for non-specific pull down), and Input RNA (to control for starting transcript abundance) we identified genome-wide significant peaks (*p* < 0.1). We processed the 6789 most abundant peaks to identify high-confidence QKI targets: we utilized differential expression pipelines to identify the subset of peaks that had significantly more counts in the QKI-6 CLIP replicates (fold change > 2, FDR < 0.05) than both IgG and Input samples. This intersectional analysis conservatively identified 437 peaks from 120 unique transcripts that were used in downstream analyses (Supplementary Data 1). These included the well-studied QKI target *Mbp* (Fig. 1C,D), as well as Carboxypeptidase E (*Cpe*), which contains predicted QKI response elements (QREs): a full QBM (ACUAAY) that is within 20 bases of a second QBM half site (YAAY). Further, unbiased motif discovery^30^ in all peaks identified a motif that matched QKI’s QBM^31^ (Fig. 1E). We further confirmed a subset of targets by IP RT-qPCR in independent samples (Supplementary Fig. 2A–C). Thus, we are confident that the mRNAs discovered via CLIP are *bona fide* QKI targets across all QKI expressing cell types in vivo.

Lastly, we found that our data had significant overlap with the only publically available QKI CLIPseq study^32^. Despite this previous study being conducted in cultured myoblasts and using a pan-QKI antibody that will bind all isoforms (not just QKI-6), we found that 13% (*p* < 1.8E−140, Fisher’s exact test) of our QKI-6 peaks overlapped with the pan-QKI peaks. Thus, in different cell types which have different mRNAs transcribed, QKI will bind distinct but overlapping target sets.

We next used our data to query the binding properties and target properties of QKI-6 in the brain. First, we analyzed the genomic distribution of peaks. Generally, RBPs involved in splicing bind highly in introns while those that modulate translation, such as mediating ribosome occupancy, transport and stability of mRNAs, often bind UTRs^33^. We found that the majority (72%) of QKI-6 peaks are in the 3′UTR, consistent with a potential role in post-splicing regulation, and only a small fraction (8%) were in introns (Fig. 1F), suggesting a minimal role of QKI-6 in splicing. These findings are in contrast to a recent pan-QKI CLIP study in cultured myoblasts, in which 38% of the targets are in introns^32^. This large intronic signal is likely due to the splicing and nuclear retention functions of the nuclear isoform, QKI-5^20,32^. Indeed, a recent QKI-5 specific CLIP experiment in E14.5 mouse brain, where QKI-5 is highly expressed in neural stem cells, showed 41.6% of binding sites in introns^28^. Recent work in *C. elegans* emphasized the importance of 5′UTR QKI motifs^34^, yet we found very few (<1%) QKI-6 peaks in the 5′UTR of mouse brain transcripts (Fig. 1F), consistent with in vitro studies that identified only 2% of pan-QKI target peaks are in 5′UTRs^32^. Thus, with its strong enrichment on 3′UTRs, QKI-6’s binding pattern appears poised to enable translation regulation rather than splicing.

**Fig. 2:**
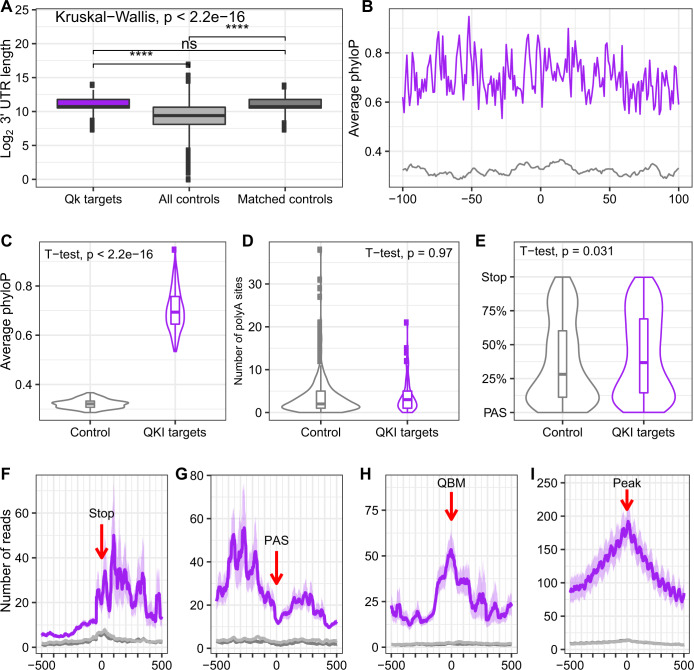
QKI-6 binds conserved UTR regions in a cooperative manner. **A** UTRs bound by QKI-6 are significantly longer than average UTRs. *N* = 574 Qk targets, *n* = 39,337 all controls, and *n* = 574 matched-length controls. Rightmost plot: matched-length controls selected for **B–G**. The significant levels represent Mann–Whitney test by comparing to UTRs bound by QKI-6. The *p* values of comparing all controls UTRs to QKI-6 bounded UTRs, matched-length controls UTRs to QKI-6 bounded UTRs, and matched-length controls UTRs to all controls UTRs are <2.2e−16, 0.68, and <2.2e−16, respectively. **B** Conservation levels (PhyloP) of 200 bp flanking QKI-6 bound sites reveal QKI-6 bound sites are more conserved than random sites in matched-length controls. **C** A violin plot illustration of the same data showing the distribution of conservation across all targets (*p* value < 2.2e−16). *N* = 200 bp flanking QKI-6 bound sites and *n* = 200 bp flanking random sites in matched-length controls. **D** A violin plot for number of PolyA sites near QKI-6 peaks reveals they are not enriched for PolyA sites (*p* value = 0.97). *N* = 141 unique 3’ UTR for Qk targets and *n* = 547 unique 3’ UTR for matched-length controls. **E** Examining the QKI peak location relative to the length of the UTRs (scaled to 100% of distance between PAS and stop codon) shows QKI-6 binding tends to be closer to stop codons (*p* value = 0.031). *N* = 426 3’ UTR for Qk targets in which QKI-6 bound sites are between their nearest PAS and stop codon and *n* = 307 3’ UTR for matched-length controls in which random sites are between their nearest PAS and stop codon. **F** Metagene of QKI-6 shows binding is elevated at stop codon, with flanking peaks, and extends substantially, especially 3’. **G** Metagene of QKI-6 shows binding is elevated at PAS, with flanking peaks, and extends substantially, especially 5’. QKI-6 binding at any (**H**) QBM or (**I**) peak extends to flanking sequence. Here, and for all box-and-whiskers plots in the manuscript, the center line represents the median, the body of the box goes from the first quartile to the third quartile (IQR), the whiskers represent 1.5x IQR from the upper or lower quartile, and points represent values that go beyond 1.5 IQR. The *p* values represent two-sided Student’s *t* test. In **B–E**, *p* values represent two-sided Student’s *t* tests. In **F–I**, ribbons represent the 95% confidence interval of the mean calculated using 1000 bootstraps. Purple = QKI-6 CLIP, light gray = Input, dark gray = IgG.

To better understand potential roles for QKI-6, we then studied the attributes of these mRNAs and the pattern of binding within their 3′UTRs. Examining UTR length distributions, it was clear that QKI-bound UTRs are on average longer than randomly selected UTRs from the genome (Fig. 2A), suggesting an evolution for greater potential for sequences mediating regulation. To test this, we selected a set of random length-matched and brain-expressed UTR sequences for controls and examined the evolutionary conservation and relative binding positions of QKI. We observed that QKI-bound sites were more highly conserved than random UTR sites (Fig. 2B, C). Examining the pattern of binding within the UTR, there seemed to be an enrichment near the stop codon, with a pattern towards peaks flanking it on either side, as well as a tendency for binding 250 bp upstream of PolyA signal (PAS) sequences. However, there was no difference in the number of PASs between QKI targets and controls, suggesting QKI does not have a general role in alternative polyadenylation (Fig. 2D). In length-normalized analyses, there were peaks near both the PAS and the stop codon (Fig. 2E, G), suggesting some interaction with both features. Complimentary to our de novo motif discovery (Fig. 1D), when we examined average read-depth around predicted QBMs in the CLIP targets, binding was clearly enriched at these motifs (Fig. 2H). Interestingly, QKI binding appears to extend from this primary high-affinity interaction to flanking nucleotides, particularly downstream. In addition to having multiple RNA interacting domains in each protein, QKI is known to homodimerize^11,35^. Thus, we speculate that this could be due to cooperativity across QKI molecules: initial binding to a high-affinity motif and homodimerization might spread binding for hundreds to thousands of nucleotides along the 3′UTR. This could also enhance folding of the UTR to bring other parts of the mRNA in contact with the same molecule: head-to-head binding of QKI molecules is thought to strongly fold RNA when binding two sites on the same molecule^35^. Finally, examining all peaks, regardless of QBM-presence, revealed a similar pattern of binding spread (Fig. 2I).

**Fig. 3:**
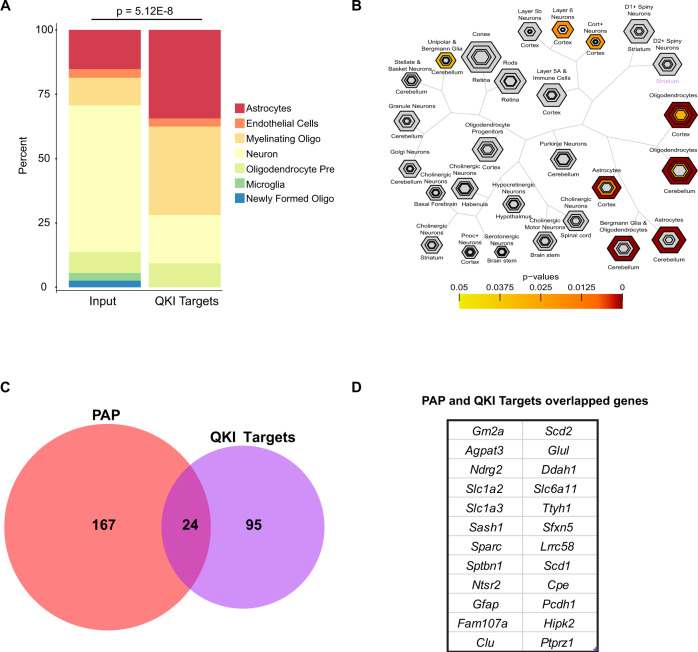
QKI-6 targets membrane protein transcripts in astrocytes. **A** QKI-6 targets are significantly enriched in transcripts from astrocytes compared to Input transcripts. *P* value represents two-sided Fisher’s exact test. Cell-type specific data is FPKM from ref. ^21^ (brainrnaseq.org). **B** Unbiased CSEA analysis^36^ shows QKI-6 targets are most strongly enriched in transcripts enriched in astrocytes and oligodendrocytes. Hexagons represent list of genes enriched in each cell type going from pSI threshold to include larger but less stringent gene lists (in this cell type and a few others) to thresholds for the most stringent subsets (smallest, central hexagons). Fisher exact testing is used to establish *p* value for each CLIP target and each gene list at each threshold and corrected by Benjamini–Hochberg for the number of cell types (color bar). Results consistent across more than one threshold hexagon per cell type are viewed with greater confidence. **C** Venn diagram of overlapping genes from PAP-TRAP^29^ and QK CLIP targets. **D** List of overlapping genes in **C**.

Next, we sought to examine the role QKI-6 has specifically in astrocytes. QKI is documented as glial-expressed^16^, however its function has primarily been investigated in oligodendrocytes. To determine the relative role in astrocytes, we assessed the percentage of QKI-6 targets highly expressed in different neural cell-types. Using Cell-type Specific Expression Analysis (CSEA)^36^, we mapped the distribution of QKI-6 targets to cells in the brain (Fig. 3A). Not surprisingly, we found that 34% of targets came from myelinating oligodendrocytes as QKI expression is dramatically upregulated in these cells compared to oligodendrocyte precursors^37^. This is consistent with our analysis of QKI-bound targets using Gene Ontology, where we identified enrichment of myelin-associated transcripts (Fig. 1D, Supplementary Data 2). However, when examining the CSEA data (Fig. 3B), astrocyte-specific transcripts represented an equal percentage of QKI-6 targets as myelinating oligodendrocytes, emphasizing that QKI may play an equally important role in translation regulation in astrocytes.

Within astrocytes, we have previously defined a set of transcripts enriched on ribosomes in the peripheral astrocyte process (PAP) and thus represent transcripts likely undergoing translational regulation either to control translation efficiency or localization^29^. To test whether QKI-6 may indeed be playing a role in regulating these transcripts, we compared our CLIP data to our prior analysis. We found that 12.6% of PAP-enriched mRNAs are also QKICLIP targets (vs 4% of Input genes, Fisher’s Exact Test (FET) *p* < 2.2E−16). Together, these data define QKI-6 targets, in vivo, and indicate a significant proportion of these targets are found in 3′UTRs of astrocyte-expressed transcripts. Furthermore, the significant overlap with PAP-enriched transcripts suggests a role for QKI in regulation of a subset of these transcripts in astrocytes.

### CRISPR-TRAPseq of QKI in astrocytes shows regulation of ribosomal association of direct targets

**Fig. 4:**
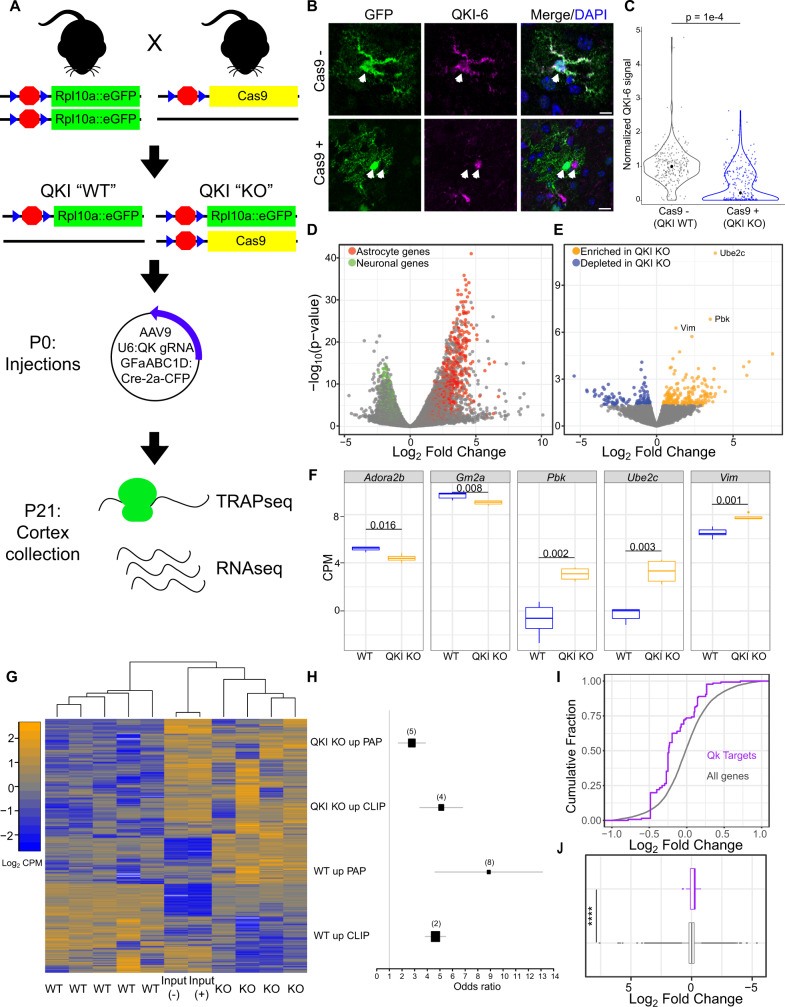
CRISPR-TRAPseq-mediated QKI deletion in maturing astrocytes alters target transcripts. **A** Cartoon strategy of QKI knockdown in cortical astrocytes. Brain icon is used under Creative Commons Attribution 4.0 International License (https://creativecommons.org/licenses/by/4.0/). **B** Transduced CFP-positive cells (detected by GFP antibody) from GFAP:CFP QKI gRNA-injected mice (Cas9−(top) and Cas9+(bottom)) reveal loss of QKI-6 immunoreactivity in Cas9+ animals, but not in Cas9− animals or in non-transduced adjacent cells. Scale bar is 20 µm. **C** Quantification of QKI pixels in CFP+ cells across genotypes reveals median 79% reduction in Cas9+ cells. Total pixel number per cell was normalized to WT littermates’ values. Median value within genotypes is represented as large black circle. *P* value represents results of a linear mixed model to account for random effects of individual animals. *N* = 270 WT, and 280 Cas9+ cells from 3 WT animals and 8 Cas9 animals. Source data are provided as a Source Data file. **D** Volcano plot showing mRNA enriched by TRAP from GFAP-Cre gRNA transduced P21 astrocytes is highly enriched in previously defined markers of astrocytes (red) and depleted in markers on neurons (green)^56^, confirming method. P-values are generated from likelihood ratio tests (LRT) in this contrast using edgeR.** E** TRAP analysis identifies transcripts with significantly altered ribosomal association in vivo following QKI mutation (blue, increased in WT, yellow increased in QKI KO. *P* values are generated from likelihood ratio tests (LRT) in this contrast using edgeR.** F** Individual genes with altered ribosomal association following QKI deletion. Data are represented by boxplots (center = median, minima and maxima represented by whisker ends, and box bounds representing the 25th and 75th percentiles of the data). The *p* value in each plot represent two-sided Student’s *t* test. **G** Heatmap of all TRAP and Input samples showing transcripts with altered ribosomal association. **H** Forest plot illustrates overlap between QKI responsive transcripts, CLIP genes, and locally translated genes as defined^5^. The odds ratio represents one-sided Fisher’s exact test. The whiskers represent 95% confidence interval for the odds ratio, and the number of genes in each group are indicated in parentheses above the bars. **I** Cumulative distribution function (CDF) and boxplot (**J**) of CLIP targets (purple) expressed in astrocytes show a decrease in QKI CLIP target transcript levels after QKI deletion (two-sided Student’s *t* test, *p* value = 2.9e−12). Data are represented by boxplots (center = median, minima and maxima represented by whisker ends, and box bounds representing the 25th and 75th percentiles of the data).

While QKI target binding cannot be conducted in a cell-type-specific manner, we did want to confirm a functional role of QKI specifically in astrocytes, and validate predicted astrocytic CLIP targets in vivo with an independent approach. RNA binding proteins can have multiple roles on their targets, including alteration of translation initiation, mRNA localization, and transcript stability. As any of these three will eventually have consequences on the amount of mRNA bound to ribosomes, we designed our approach to quantify ribosomal association by mRNAs after QKI deletion in maturing astrocytes in vivo. Because QKI knockouts are not viable^12^, and the most commonly used mouse model (quaking viable, QK^v^) is an enhancer deletion which impacts only oligodendrocytes^16^, we developed a strategy, CRISPR-TRAPseq, to delete QKI in astrocytes and assess changes in ribosomal association in these cells. We designed the deletion to be mosaic – impacting a subset of GFAP+ cells in otherwise normally developing brains - to mitigate most non-cell-autonomous effects from mutation. Specifically, we generated knock-in mice containing a loxP-stop-loxP (LSL) Cas9 allele in the Rosa locus that can be expressed under a Cre-inducible promoter (Supplementary Fig. 3A–C). We then crossed heterozygotes of this line with mice homozygous for the LSL-Translating Ribosome Affinity Purification (TRAP) construct in the same locus (Fig. 4A). Next, we transcranially injected entire litters of perinatal pups with an AAV expressing Cre and CFP-myc under the control of a GFAP promoter, along with gRNAs targeting QKI under a second promoter (U6), to generate mosaic deletions. Thus, subsequent to GFAP:Cre activity, *all* transduced GFAP positive cells will express the TRAP allele, allowing for cell-type-specific enrichment of tagged ribosomes. Further, 50% of these animals will also express Cas9, thus mutating QKI through nuclease cleavage and non-homologous end joining, inducing point mutations and indels into the coding sequence. A key advantage of this approach, as compared to virally deleting QKI in a normal Aldh1L1 bacTRAP mouse where all astrocytes express the TRAP allele, is that here *only* the cells that turn on Cas9 also turn on the TRAP allele. Thus, TRAP enrichment allows one to very specifically assess mutant cells in the context of an otherwise wildtype brain.

First, we confirmed that our Cas9 strategy for gene disruption was effective by immunofluorescence assessing QKI-6 protein (Fig. 4B). In P21 animals, we saw robust expression of CFP within cells that exhibited typical astrocyte morphology. Immunofluorescence revealed that cells showed median 79% loss of QKI-6 protein in Cas9+ animals (henceforth referred to as QKIKO), significantly different from Cas9- littermates (Fig. 4C). Across animals, we found that a mean of 48.8% ± 23% of QKIKO cells showed less than 10% of QKI-6 signal compared to their WT littermates (Supplementary Fig. 3D). We then conducted TRAPseq on additional animals of each Cas9 genotype. While yields were too low to conduct PAP-TRAP to examine local translation, standard TRAP yielded high quality (RIN > 7.7) RNA across replicates, which was then sequenced to a depth of >27 M reads/sample. Cas9 was detectable in the RNAseq, confirming the genotype of each animal. Further, comparison to total RNA ‘Input’ controls reveal a robust enrichment of known astrocyte-expressed genes, and depletion of neuronal genes (Fig. 4D), indicating the approach was yielding RNA from descendants of perinatal GFAP expressing cells (e.g., astrocytes). Indeed, this relative enrichment of positive control transcripts, and depletion of negative control transcripts was comparable to our prior experiments utilizing our Aldh1L1 bacTRAP animal, validating the efficacy of the method (Supplementary Fig. 4).

Then, we examined the consequences of QKI mutation on mRNA in astrocytes. Given that the deletion was occurring across a period of postnatal astrocyte maturation, and that the mutation in QKI will impact all isoforms of QKI protein, we expected the resulting profile to be a mix of effects of loss of QKI-5, -6, and -7. Overall, we see clear effects of QKI mutation on ribosomal association with 241 nominally upregulated and 115 nominally downregulated genes (Supplementary Data 3, Fig. 4E, F). Consistent with TRAP sensitively assessing the mutant cells, these effects were largely not apparent in the Input RNAseq, demonstrating the need for the targeted enrichment (Fig. 4G). Examining the regulated transcripts, in spite of this being a mix of direct and indirect effects, a small but significant fraction of 3′UTR CLIP targets of QKI-6 are significantly modulated (OR = 5.07, FET *p* < .0018), especially those found in astrocytes (OR = 8.8 FET *p* < .0016). Both up and down regulated transcripts had significant overlap with CLIP targets and those previously described as being translated in PAPs (Fig. 4H), with the KO losing ribosomal association of transcripts from PAPs. Looking at TRAP-seq across all annotated CLIP targets, there was no obvious consistent change, though many of those genes are not expressed in astrocytes. Therefore, subsetting to only CLIP targets expressed in astrocytes, we saw that transcripts from QKI-mutants exhibited a small but significant decrease in mRNA as measured by TRAP (average 10% decrease, *p* < 2E−12, *t*-test) (Fig. 4I, J), validating the CLIP results. These data are consistent with a role for QKI normally in increasing transcript stability and/or ribosomal association of the majority of its direct targets in astrocytes, and is of a similar magnitude and direction of effect to what was previously seen in HEK cells^35^. CLIP targets bound in 5′ UTR, though rare (*n* = 31), had a similar effect (15% decrease, *p* < 9.1E−5). In contrast, rare targets with QKI-6 binding in introns did not show a consistent direction of effect. It is worth noting the resultant impact on the final protein and mRNA levels following QKI deletion could be mixed as QKI may have distinct roles across its varied targets, or that deletion of all nuclear and cytoplasmic QKI isoforms across postnatal development results in a complicated mix of splicing, translational, and/other indirect effects within the cells. For example, *Pbk* and *Ube2c* genes do not appear to be QKI-6 CLIP targets in normal P21 brain, but are upregulated on QKI loss (Fig. 4F).

As our gRNA targets all isoforms, QKI deletion could also feasibly impact splicing. In a transcript isoform level analysis, we detected 949 transcripts with differential expression arising from 833 genes, including all of those found with the gene level of analysis (Supplementary Data 4). For the 74 genes with more than one isoform changing, 40 had isoforms changing in reciprocal directions, which could plausibly represent targets of alternative splicing by QKI-5. Indeed, QKI is known to autoregulate its own splicing at specific exons, and we do see a 3-fold upregulation of a specific isoform of QKI in our data. This isoform (ENSMUST00000097414) has the C-terminal amino acid sequence of QK6, but due to a UTR splicing event, the last exon of QK5 is included though out of frame. Based on standard NMD rules, having a splice junction downstream of a stop codon should lead to a very unstable transcript. This suggests loss of QK protein levels might be either stabilizing this transcript somehow, or promoting this aberrant splicing event. Both are plausible as QK extensively binds its own transcript in our CLIP data.

### Loss of QKI alters astrocyte transcriptional maturation

**Fig. 5:**
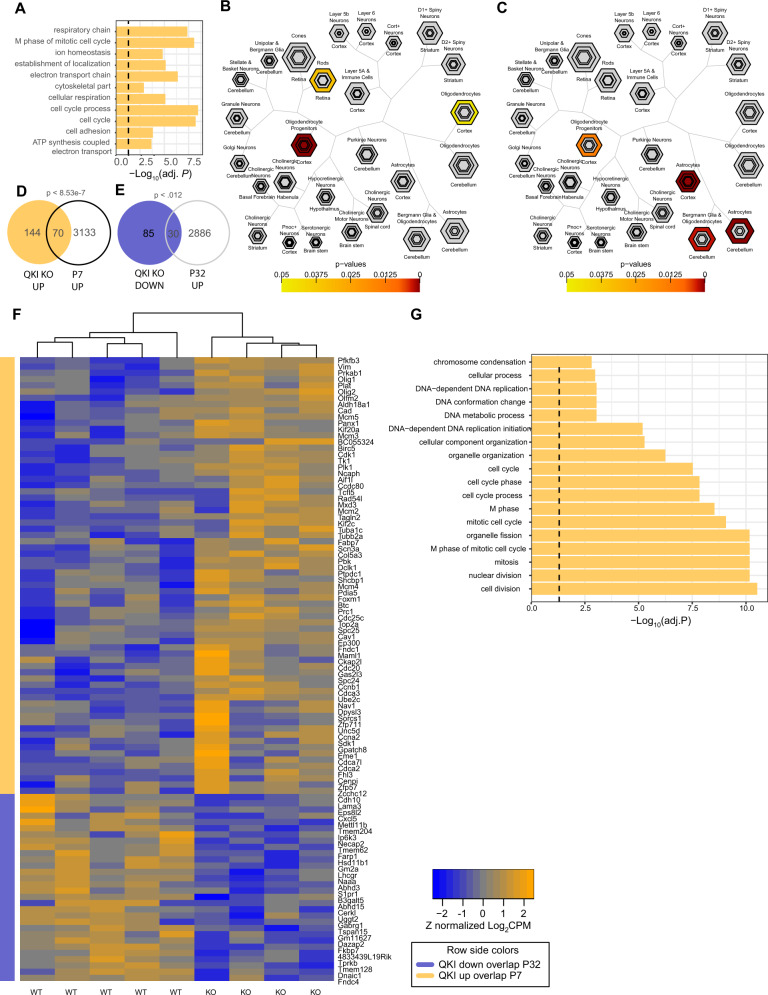
QKI deletion delays a subset of astrocyte transcript maturation. **A** Gene Ontology analysis of biological processes enriched by QKI loss reveal increased mitochondrial and cell proliferation. **B** CSEA of transcripts increased when QKI is deleted are disproportionately found in oligodendroglial progenitor cells. Cell-type-specific data is FPKM from ref. ^21^ (brainrnaseq.org). **C** CSEA of transcripts decreased when QKI is deleted are disproportionately found in adult astrocytes. Cell-type-specific data is FPKM from ref. ^21^ (brainrnaseq.org). **D** Transcripts increased when QKI deleted contain proportionally more P7 astrocyte transcripts. **E** Transcripts decreased when QKI deleted contain proportionally more P32 astrocyte transcripts. **F** Heatmap of transcripts both modulated by QKI mutation and differentially expressed between P7 and P32. **G** The 70 transcripts from **D** are highly enriched in cell cycle-related biological processes. The 30 transcripts from **E** did not significantly map to a defined biological process.

Analysis of transcripts altering mRNA by TRAPseq can indicate the function of QKI in GFAP positive cells during postnatal astroglial maturation. Conducting a pathway analysis on the genes upregulated in CRISPR-TRAPseq by loss of QKI indicated a role in cell cycle, especially M-phase (*p* = 1.4E−8, FDR corrected), and a variety of mitochondrial proteins involved in electron transport chain (*p* = 4.1E−7, FDR corrected) (Fig. 5A, Supplementary Data 5). The relation to cell cycle in particular could be consistent with a role for QKI in astrocyte maturation, similar to what was previously reported for oligodendrocyte maturation^17^. For example, the mitosis activated kinase Pbk^38^ is upregulated with QKI mutation. Thus, QKI may be required for decreasing proliferation and increasing fate choice, as GFAP positive progenitors from P1 mice have been shown to contribute to both astrocyte and oligodendroglial lineage^39,40^. To first test this hypothesis, we used CSEA analysis of CRISPR-TRAPseq targets (Fig. 5B, C). We saw that QKI mutant astrocytes showed more expression of genes associated with ‘OPC’ cells, a proliferating glial progenitor with potential to turn into both mature astrocytes and oligodendroctyes^41^. This result would be consistent with either a disruption of astrocyte maturation, or potentially QKI loss leading to a shift in fate towards an OPC lineage. However, immunofluorescence for the OPC marker Ng2 ruled out the second possibility (Supplementary Fig. 5). Prior work has shown QKI knockout in embryonic neural stem cells prevented their differentiation^28^. Thus, it is plausible that QKI mutation in postnatal GFAP+ cells may influence maturation of astrocytes as well. To further test this hypothesis, we generated a list of genes enriched by TRAP in immature (P7) vs. mature (P32) astrocytes^15^ and compared them to transcripts altered by QKI mutation. We found that indeed deleting QKI resulted in prolonged P7 gene expression and decreased P32 gene expression (Fig. 5D, E), consistent with QKI being necessary for astrocyte transcriptional maturation at a subset of transcripts. Over the course of postnatal astrocyte maturation, astrocytes become more morphologically complex and extend their branches to infiltrate the neuropil^42,43^. Therefore, we asked if transcriptional maturation corresponded with astrocytic growth. We quantified astrocytic neuropil infiltration volume (NIV) as a proxy for morphological maturation. Interestingly, we found no significant difference in astrocytic NIV between QKI+ and QKI− astrocytes (Supplementary Fig. 6) indicating that genes directing morphological maturation are not targets of QKI in astrocytes. Overall, our data are consistent with QKI deletion resulting in a mix of direct and indirect cell-autonomous effects, with contributions of all QKI isoforms, and a final consequence on suppressing astroglial maturation transcriptionally, but not for neuropil infiltration.

## Discussion

Here, we define targets of QKI-6 in vivo using CLIPseq on mouse forebrain. We initially focused on what QKI-6’s binding pattern indicates about function. First, these data reveal, similar to in vitro studies^32^, that QKI-6 target peaks generally reside in 3′UTRs, supporting a role of QKI in translational control^44^ and mRNA stability^45^. This finding is in contrast to previous pan-QKI CLIPseq studies in which introns represented a large percentage of signal^28,32^, thus highlighting a significant difference across QKI isoform binding. Consistent with binding having important biological roles, bound regions were more conserved and contained enrichment of QBMs. Second, peaks were enriched near stop codons and PolyA signals, and while enriched in QBMs, many sites did not contain them. Further binding was elevated even hundreds of nucleotides from the QBM/peak of highest binding. As QKI is known to homodimerize^11,35^, this spreading binding could be consistent with a cooperativity model where a high-affinity site recruits QKI, which then facilitates additional binding at lower-affinity sites nearby, either through the additional domains in the same molecule, or across dimerized QKI complexes. Of particular interest is the pattern of dual peaks flanking stop codons (Fig. 2F). As QKI homodimers have been thought to encourage looping of RNA^35,46^, such looping across the stop codon could have important consequences for translational elongation or termination, and could be of interest for future studies.

Motivated by curiosity about translational regulation in astrocytes, we next focused on the significant subset of CLIP targets known to be expressed in them. Our data showed an enrichment of QKI-6 targets in PAP-enriched transcripts^5^, consistent with QKI-6 having some role in regulation of ribosomal association in astrocytes. Perhaps similar to its documented role on MBP mRNA, this could be related to nuclear export or pausing during transport, rather than mediating localization per se^20^. QKI-6-bound transcripts include regulators of synapse numbers (*Sparc*, *Sparcl1*), neurotransmitter uptake (*Slc1a2, Slc1a3*), and astrocytic cytoskeleton (*Gfap*). Interestingly, *Gfap* is a known QKI target in human astrocytes^25^, and other cytoskeletal genes are QKI-regulated in oligodendrocytes^18^, leading to a hypothesis that QKI controls astrocyte development and/or astrogliosis in injury and disease.

Testing this idea, however, represented a challenge. The most elegant approaches for defining gene function are deletion studies. However, astrocyte maturation and synaptogenesis are in vivo processes occurring over the first weeks of postnatal life. Yet, germline deletion of QKI is embryonic lethal^47^. While the Cre-Lox system is sometimes used to circumvent these issues, during the course of these studies floxed QKI mice were not available. Even then early postnatal studies remain challenging – constitutive astrocyte Cre lines are also expressed in radial glia in embryonic brain^40,48^, where QKI has clear roles in neural stem cells^28^. It is likely brain development would be substantially perturbed obscuring any of the later cell-autonomous effects in maturing astroglia. Some inducible Cre lines for astrocytes exist^49^, however, perinatal tamoxifen injection both dramatically alters sexual differentiation – an effect previously reported in rats that we replicated in mouse *-* and leads to substantial rates of delayed lethality: we saw 43% mortality before weaning across 57 pups injected with >100 µg of tamoxifen before P7. Furthermore, an ideal approach would perturb relatively few cells to limit non-cell-autonomous effects on development overall, and would be coupled to a method to specifically yet comprehensively profile cell-autonomous effects. To this end, we applied a CRISPR-TRAPseq approach, wherein sparse gene deletion in specific cell-types, mediated by postnatal gene editing, is coupled to TRAP for specific profiling of targeted cells. We utilized this method to define the consequences of QKI mutation on transcriptome-wide ribosomal association in astrocyte development. We saw alterations due to both changes in splicing as well as transcript levels on ribosomes, and these consequences included both direct (CLIP-target) and indirect effects within astrocytes. Generally, there was a tendency for QKI loss to decrease ribosomal association of QKI-6 CLIP targets, perhaps reflecting a loss of transcript stability as seen in cell line QKI mutant studies^35^. This result could be consistent with QKI stabilizing these mRNAs, increasing translation initiation, or perhaps stalling mRNAs on ribosomes. Nonetheless, gestalt analysis across altered transcripts argues that QKI is required for a specific aspect of astrocyte transcriptional maturation. This is consistent with a very recent report showing QKI deletion in nestin-positive stem cells disrupts specification of stem cells to glial and astrocyte lineages^50^. Thus, across the two studies, it appears QKI might be necessary both for early differentiation of glial stem cells prior to GFAP expression, as well as later transcriptional maturation of astrocytes as shown here.

What is the specific function of the genes targeted by QKI during astrocyte maturation? A Gene Ontologies analysis of the specific subset of maturing astrocyte genes that are lost with QKI knockdown revealed an enrichment of glycoproteins (*p* < 2.5E−6, *p* < 0.02 after FDR correction), with trends in lipid metabolic processes and signal peptide-containing genes. This suggests QKI is regulating translation of proteins with a role at or near the cell surface, consistent with a hypothesized role in regulating some locally translated transcripts^29^. Indeed, we noted an enrichment among QK targets of our previously PAP-defined transcripts, and this included some of the maturation regulated genes (*GM2a*, *Sfnx5*, and *Sptbn1*). Of particular interest among these is the GM2 ganglioside activator (*GM2a*), which activates lysosomal breakdown by beta-hexomaminidase A of GM2 gangliosides, the toxic byproduct that aggregates in Tays-Sachs and other gangliosidoses. Indeed, loss of GM2a itself can lead to the AB variant of this disease^51^. As these gangliosides are prevalent on neuronal membrane, the upregulation of this key lysosomal regulator during a period of astrocyte maturation that covers synaptic pruning, suggests QKI might dynamically regulate aspects of local lysosomal function related to synaptic pruning by astrocytes and the subsequent clearance of this breakdown product. Indeed, a very recent report defined a similar role for QKI in microglia in clearance of myelin debris after experimentally induced demyelination^52^.

Finally, the CRISPR-TRAPseq approach proved useful in systematically defining the consequences of QKI deletion in sparse astroglia in a normally developing brain, and we believe it will be similarly useful for other contexts. For example, swapping promoters will allow targeting of different cell-types, as could electroporation at defined time points, provided yields were sufficient. Likewise, gRNAs could target any gene, though RBPs and transcription factors will be especially amenable to TRAP-based readouts. It was also encouraging that TRAP RNA appeared similar in purity with standard bacTRAP (Supplementary Fig. 4). Thus, CRISPR-TRAP should be an efficient means to define gene functions in vivo. It is important to note that not all cells showed deletion (Fig. 4C, Supplementary Fig. 3D), and there was surprising animal to animal variability. We suspect remaining cells had silent or missense mutations that disrupted further gRNA binding without altering QKI expression, or there was some perdurance of QKI RNA, perhaps influenced by QKI’s autoregulation^32^. Adding multiple exon-targeting gRNAs to each vector could remove this vestigial expression. Also, for this study, we delivered Cas9 and TRAP via Rosa alleles and this allowed for a well-controlled littermate design. However, this design can be implemented via similar approaches delivering these components exogenously (e.g., electroporation or AAV) enabling access to other species. Likewise, existing TRAP and Cas9 FLEX cassettes would enable access to the catalog of available Cre mice, further broadening applicability of the approach. Here, focusing on astrocyte translation, we used this approach as a follow up and validation to a comprehensive CLIP analysis, allowing us to confirm impact on CLIP targets in vivo, and describe a role for QKI in transcriptional astrocyte maturation at a subset of transcripts.

## Methods

### Mice

All procedures were approved by and performed in accordance with the guidelines of the Institutional Animal Care and Use Committee at Washington University in St. Louis. Mice were maintained in standard housing conditions with food and water provided *ad libitum*, and crossed at each generation to wildtype C57BL/6J mice from Jackson labs.

#### Generation of Cas9 Rosa mice

To generate a Cre-dependent Cas9-expressing mouse line, we engineered Cas9 into a targeting vector containing Rosa26 homology arms (~1 kb, PCR’s from genome), flanking a CAG enhancer, Lox-Stop-Lox cassette (adapted from the Allen AI9 constructs, Addgene Plasmid #22799), with 3xFlag-NLS-Cas9-NLS cloned downstream of the stop cassette. The Flag tag is in frame to facilitate confirmation of protein expression. Targeting vector was sequence confirmed then purified for injection into C57BL/6 X CBA F1 hybrid oocytes in conjunction with mRNA coding for a pair of custom TALENs designed to target the mouse ROSA26 locus between the homology arms using the ZiFit targeter (http://zifit.partners.org) and ROSA26 TALENs binding sites as follows:

1. 5′ ROSA26 TALEN binding site: 5′ tccctcgtgatctgcaactcc 3′
2. 3′ ROSA26 TALEN binding site: 5′ gggcgggagtcttctgggca 3′

The TALEN kit used for TALE assembly was a gift from Keith Joung (Addgene kit # 1000000017). DNA fragments encoding ROSA TALEN repeat arrays were cloned into plasmid pJDS71. ROSA TALENs plasmids were linearized for in vitro transcription with EcoRI and TALENs RNA was synthesized using the mMessage mMachine T7 Ultra kit (Ambion) and purified with Megaclear columns (Ambion). The cassette of interest was introduced into the mouse genome via pronuclear injection of in vitro transcribed TALENs RNA and ROSA donor DNA. Founders with correctly targeting mutant alleles were identified using long-range PCR with ROSA specific oligos outside of the homology arms and insert specific oligos. Founders may have been mosaic with multiple mutant alleles and thus were bred to WT to generate heterozygous F1 offspring. Analysis of F1 offspring via long-range PCR confirmed germline transmission of the correctly targeted allele.

Such progeny of founder MR04 were then bred to a ubiquitous germline Actin-Cre mouse line to confirm Cre-dependence of Cas9 expression (Supplementary Fig. 3, Full blot images can be found in the Source Data file). Subsequent generations of Cre- mice were backcrossed to C57BL/6J wildtype mice.

Cas9 efficacy in gRNA-mediated gene targeting was confirmed via loss of QKI protein subsequent to delivery of QKI targeting guide RNAs in Cre-expressing transduced astrocytes in vivo (Fig. 4A, B). Mouse lines and other unique biological reagents are available to academic researchers upon reasonable request.

#### CLIP

(I) Dissection, lysis and RNAse treatment: Five P21 C57BL/6J mice were anesthetized by inhalation of isoflurane. Mice were decapitated and brains were quickly dissected and moved to ice-cold PBS. The forebrain was removed and dropped into liquid nitrogen in a ceramic mortar and then powdered with a ceramic pestle. Powdered brains were kept in 6 cm petri dishes on dry ice until cross-linking. UV cross-linking was performed in a Stratalinker, at 400 mJ/cm^2^, 3 times with agitation of the powder between runs to ensure even cross-linking. One sample was not cross-linked, as a control (No XL). Powdered brains were then resuspended in 1 mL ice-cold 1X Lysis Buffer (50 mM Tris-HCl pH 7.4, 100 mM NaCl, 1%NP-40, 1X Roche c0mplete EDTA-free protease inhibitor, 1U/μL recombinant RNAsin (Promega), 10 mM Na_3_VO_4_, 10 mM NaF) and homogenized with a drill (power 14) 10 times. Samples were incubated on ice for 5 min then immediately treated with appropriate concentration of RNAse I_f_ (NEB) for 3 min at 37 °C at 1200RPM. Samples were centrifuged at 20,000 × *g* at 4 °C for 20 min. 2% of the supernatant was kept for Input sample, the rest of the supernatant was kept for immunoprecipitation.
(II) Immunoprecipitation: Per IP, 6 μl of QKI-6 antibody (Millipore #AB9906) or total Rabbit IgG (Jackson Immunoresearch #711-005-152) was added to 72 μl of M280 Streptavidin Dynabeads (Invitrogen) and 10 ug of Biotinylated Protein G and incubated for 1 h at room temperature with rotation. Beads were subsequently washed 5 times with 0.1% BSA (Jackson Immunoresearch) before mixed with supernatant. Immunoprecipitation proceeded for 2 h at 4 °C with rotation, after which beads were washed with 1X High Salt Wash Buffer (50 mM Tris-HCl pH 7.4, 350 mM NaCl, 1%NP-40 and 1 unit/μl Promega recombinant RNAsin). In the last wash, beads were split for RNA extraction (98%) and western blot (2%).
(III) RNA extraction: Beads were resuspended in 1X LDS Bolt Non-Reducing Sample Buffer, heated for 10 min at 70 °C then run out on a 4–12% Novex NuPAGE gel in 1X NuPAGE MOPS buffer with a protein ladder (Bio-Rad). The gel was transferred to a 0.2 μm PVDF membrane for 2 h at 200 mA at 4 °C. Individual lanes were cut with a razorblade between 37 and 50 kDa, to obtain, on average, ~100 base length fragments. Membrane strips for each sample were digested in 200 μl of 100 mM Tris-HCl pH 7.4, 50 mM NaCl, 10 mM EDTA, 1% Triton-X 100 and 32 units Proteinase K (NEB) at 37 °C for 1 h, with shaking. 200 μl of 7 M Urea was added to each sample for an additional 20 min at 37 °C, with shaking. After, 400 μl of Acid-Phenol:Chloroform (pH 6.5) was added to each sample, incubated for 5 min then spun at 10,000 × *g* for 7 min at room temperature. The aqueous layer was kept and subsequently cleaned up on the Zymo RNA Clean and Concentrator 5. Quality and quantity of RNA were assessed on an Agilent TapeStation.
(IV) Sequencing Library Preparation: Because yields of RNA after CLIP are extremely low (picograms), all RNA harvested was used for library prep. We used direct ligation of an RNA adapter to the harvested RNA after initial dephosphorylation (NEB Antartic Phosphatase) of free ends. RNA was purified using MyONE Silane Dynabeads (Thermo Fisher). Direct ligation of the A01m adapter (Supplementary Table 1). was added at a final concentration of 1 μM using T4 RNA ligase (Enzymatics). Reverse transcription of purified A01m-RNA was carried out using the AR17 primer (ACACGACGCTCTTCCGA) at a final concentration of 1 μM, using SuperScript RT III First Strand Synthesis System (Thermo Fisher). RNA and remaining primers were subsequently destroyed with ExoSAP-It (Affymetrix), heat and NaOH. Lastly, a final ligation to cDNA was done to add unique molecular identifiers (allowing removal of PCR duplicates computationally). The Rand103tr3 Adapter (Supplementary Table 1) was added at a final concentration of 2 μM, using T4 RNA ligase (Enzymatics). PCR (20 total cycles) was then carried out using the Q5 Ultra II Q5 Master Mix (NEB) to amplify final libraries using universal Illumina primers, with sample indexing primers, for sequencing. Detailed protocol is available from the Dougherty Lab upon request.
(V) Peak Calling and Differential Expression: Peaks were called across the genome using Piranha^53^ v1.2.1. The mouse genome (mm10) was binned into 50 nucleotide non-overlapping windows. Peaks were called under the genome windows on a merged.bam file from all QKI IP samples. Significant peaks were determined by genome-wide FDR *p* value < 0.05. Count analysis was performed using Subread^54^ v1.5.3, for each sample file. Differential expression was performed using the edgeR package in R. Per edgeR instructions, only peaks with a minimum of five counts (CPM of 41) in two or more samples were analyzed. High-confidence QKI peaks were identified by 2-fold enrichment over both IgG IP and Input samples, with an FDR-corrected *p* value of <0.1 for discovery purposes. A control set of Input ‘peaks’ is defined as all detected RNAs in the Input matching the CPM cut off described above.

Data are available at GEO: GSE147121.

#### CLIP analysis

(I) MEME Analysis: MEME-CHiP v4.12.0 was performed in discriminative mode, using Input peak sequences as background. Default settings were applied with the exception of using 4 nucleotides as the minimum motif width.
(II) Cell-Type Specific Expression Analysis (CSEA): Analysis for unique genes from QKI or Input significant peaks was performed using the pSI package^55^. Specificity indices were determined using previously published RNA-seq data^21^. A Chi-square test was used to determine whether the distribution of cell-types in QKI or Input samples were significantly different.
(III) Gene Ontology Analysis: GO analysis was performed in Cytoscape v3.5.1 using the BiNGO plug in. Input peak genes were used as the background list. Significant biological process categories were determined by hypergeometric test with Benjamini–Hochberg correction. For analysis of CLIP targets within astrocytes, CLIP targets were subsetted to those genes enriched in astrocytes (logFC > 0.5 and False Discovery Rate (FDR) > 0.1 in TRAP vs Input comparison, below), and the background for BiNGO analysis was set to all genes enriched in astrocytes.
(IV) Analysis of sequence features of targets

CLIP Analysis: 25000 transcripts which have 3′ UTR and 3′ UTR length ≥50nt were randomly selected from all mouse (GRCm38.97) transcriptions. The 3′ UTR sequences from a subset of transcripts which have the same length distribution as the CLIP targets, as well as measurable expression in brain RNASeq data (>10 counts), were selected as control sequences. The artificial peak location of a control sequence was randomly picked within the 3′ UTR region.

The conservation scores of the CLIP targets and control sequences were retrieved from UCSC basewise conservation scores (phyloP) of 59 vertebrate genomes with mouse. The average conservation scores from CLIP targets are compared with those from control sequences.

Number of polyA sites of each sequence was counted by scanning the polyA signal (A[AT]TAAA). To demonstrate whether the peaks are closer to stop condon or polyA sites, the percentage of distance was calculated as (polyA_site pos − peak pos)/(polyA_site pos − stopcodon pos) $*$ 100 for sequences from plus strand, while (peak pos − polyA site pos)/(stopcodon pos − polyA site pos) $*$ 100 for sequences from minus strand.

Metagene plots were generated by metagene R package 2.14.0 with given alignment files from CLIP, Input and IgG. The expression level of the 1000nt region centered at stop codon, PAS, QBM and peak of CLIP were compared with those of Input and IgG, respectively.

#### CRISPR/Cas9 AAV

(I) QKI gRNA design and sequences: gRNAs for QKI was designed using the Zhang lab CRISPR design tool (crispr.mit.edu).
  Target: QKI, exon 2
  gRNA: ATGTACAATGACACGTTAAA
(II) QKI knockdown in vivo: QKI gRNA was cloned into AAV:ITR-U6-sgRNA(backbone)-pCBh-Cre-WPRE-hGHpA-ITR, a gift from Feng Zhang (Addgene plasmid # 60229). The plasmid was modified such that pCBh promoter was replaced with GFABC1D promoter, and Cre was replaced with CFP and Cre, separated by a 2a peptide. The vector was packaged with AAV9 capsid by the Hope Center for Viral Vectors at Washington University. 1 μl of high titer virus was injected into P0 lox-stop-lox Cas9 knock-in mice (Rosa26:Cas9^tg/+)^ or Cas9-negative littermates (for imaging) or progeny of (Rosa26:Cas9^tg/+^) X (Rosa26:TRAP^tg/tg^) for CRISPR-TRAPseq. Mice were anesthetized on ice for 5 min before injections and allowed to recover on heating pad for 15 min. Two microliters of 10^12^ vector genome (vg)/mL virus was injected bilaterally in the cortex, 1.5 mm from the midline in two regions: 1-mm caudal to bregma and 2-mm rostral to lambda. Injections were performed with a 33-gauge needle (Hamilton #7803–05) with a 50-μL Hamilton syringe (#7655-01). Mice were aged until P21 before being killed with CO_2_ and transcardial perfusion with PBS, followed by 4% PFA in PBS.

#### CRISPR-TRAPSeq

Six Cas9+ and six Cas9-negative littermates, all with the TRAP allele, each received intracranial injections of the QKI gRNA AAV (see above) at P1-3 and were sacrificed at P21 for CRISPR/TRAP-Seq. TRAP was performed on P21 cortex homogenates as described previously^5^. RNA quality and concentration were assessed using RNA PicoChips on the Agilent BioAnalyzer following manufacturer’s instructions. Only RNA samples with RIN scores > 7.5 were submitted for library preparation and RNA sequencing.

#### RNA sequencing and analysis

Library preparation was performed with 0.1–5 ng of total RNA, integrity was determined using an Agilent bioanalyzer. ds-cDNA was prepared using the SMARTer Ultra Low RNA kit for Illumina Sequencing (Clontech) per manufacturer’s protocol. cDNA was fragmented using a Covaris E220 sonicator using peak incident power 18, duty factor 20%, cycles/burst 50, time 120 s. cDNA was blunt ended, had an A base added to the 3′ ends, and then had Illumina sequencing adapters ligated to the ends. Ligated fragments were then amplified for 14 cycles using primers incorporating unique dual index tags. Fragments were sequenced on an Illumina NovaSeq using paired reads extending 150 bases targeting 30 M reads per sample.

Basecalls and demultiplexing were performed with Illumina’s bcl2fastq software and a custom python demultiplexing program with a maximum of one mismatch in the indexing read. Sequencing results were quality checked using FastQC version 0.11.7. Reads aligned to the mouse rRNA were removed by bowtie2 version 2.3.5. Illumina sequencing adapters were removed and the remaining RNA-seq reads were then aligned to the Ensembl release 97 primary assembly with STAR version 2.5.1a. Gene counts were derived from the number of uniquely aligned unambiguous reads by htseq-count version 0.9.1. Isoform expression of known Ensembl transcripts was estimated with Salmon version 0.8.2. Sequencing performance was assessed for the total number of aligned reads, total number of uniquely aligned reads, and features detected. TRAP performance was assessed by PCA and comparison to prior studies. Samples were excluded if TRAP failed (no enrichment of astrocyte genes compared to input). The ribosomal fraction, known junction saturation, and read distribution over known gene models were quantified with RSeQC version 2.6.2.

For gene level analyses, all gene counts were then imported into the R/Bioconductor package EdgeR5 and TMM normalization size factors were calculated to adjust samples for differences in library size. Only genes that have sufficiently large counts in the smallest group size samples were retained for further analysis using the default settings of the filterByExpr function in EdgeR. Differential expression analysis was then performed to analyze for differences between conditions. A negative binomial generalized log-linear model (GLM) was fit to the counts for each gene. Then the likelihood ratio tests (LRT) were conducted for each comparison. Similar approaches were taken for transcript isoforms, but the differential expression analysis used the R/Bioconductor package Limma with the voomWithQualityWeights function to further account for increased levels of variance at the isoform level.

P7 and P32 astrocyte transcripts were identified by differential expression analysis (*p* value < 0.05) on P7 and P32 gene counts from the cortex, hippocampus and striatum^15^. Up- and down-regulated transcripts when QKI was deleted were compared with P7 and P32 astrocyte enriched transcripts by Fisher’s exact test.

Cell-type Specific Expression Analysis (CSEA) was used to map the cell distribution in the brain of QKI-6 targets, up- and down-regulated transcripts when QKI was deleted, respectively, as described^36^. Gene Ontology analysis of biological processes was performed on the upregulated transcripts when QKI was deleted.

### Immunofluorescence

Mice were sacrificed by inhalation of isoflurane and then rapidly perfused with ice-cold 1X PBS, followed by 4% paraformaldehyde. Brains were post-fixed overnight and then cryoprotected in 30% sucrose. Sectioning was performed on a cryostat (Leica). Immunofluorescence (IF) was performed on 14 μm slide-mounted coronal brain sections in all cases. Slides were blocked in 5% Normal Donkey Serum (Jackson Immunoresearch) with 0.25% Triton X-100 in PBS for at least 30 min at room temperature. Primary antibodies were incubated overnight at 4 °C (see Table 1). Secondary antibodies (Invitrogen) were incubated at 1:1000 for 90 min at room temperature. Quantification of total pixels was performed blind to genotype and/or condition using custom macros (available upon request) for ImageJ (NIH). N (cells) is represented in the figure legends. Experiments were performed on at least 3 mice per genotype. Analysis of QKI6 knockdown efficiency was performed in R using a Linear Mixed Model to account for the random effect of individual animal.

**Table 1:** Antibodies and dilutions used for immunofluorescence.

Antibody	Catalog number/Company	Dilution
QK5, Rb	AB9904/Millipore	1:500
QKI-6, Rb	AB9906/Millipore	1:500
QK7, Rb	AB9908/Millipore	1:500
GFP, Ck	GFP-1020/Aves	1:1000
NG2, Rb	AB5320/Millipore	1:500

### Neuropil infiltration quantification

Quantification of the neuropil volume was performed as previously described^43^. Briefly, 15–20 µm Z stacks were acquired at a 0.5 µm interval on a Zeiss confocal microscope using 63× magnification and 2× zoom. Images were analyzed in Imaris (v9.5). Surfaces were reconstructed from a 13 × 13 × 10 (µm) region of interest in which no detectable neuronal cell bodies, blood vessels or major astrocyte branches were found. The default surface rendering parameters were used to control for bias in the analysis, with the exception that the minimum fragment sized was changed from 10 to 1 to capture the smallest branches.

### Western blotting

Whole mouse brains were dissected after isoflurane inhalation and rapid decapitation. Brains were homogenized (10 mM HEPES pH 7.4, 150 mM KCl, 5 mM MgCl_2_, 0.5 mM DTT, supplemented with 1 μl rRNAsin (Promega) and 1 μl Superasin (Ambion) per ml and 1 mini EDTA-free protease inhibitor tablet (Roche) per 10 ml) using a glass homogenizer and Teflon pestle with a drill. Lysates were cleared by centrifugation at 2000 × *g* for 10 min. Concentration of the supernatant was determined by BCA assay (Thermo Fisher). 50 ug of total lysate was run on 4–12% SDS-PAGE gel (Bio-Rad) and transferred to 0.2 μm PVDF membrane (Bio-Rad). Ponceau image was quantified for loading control. Membranes were blocked in 5% Milk in 1X TBS with 0.5% Tween for 3 h at room temperature. Membranes were incubated in antibody overnight at 4 °C (QKI-6, AB9906/Millipore, 1:1000). Appropriate HRP conjugated secondary antibodies were applied after washes at room temperature for 1 h. Membranes were incubated in Clarity (Bio-Rad) chemiluminescence reagents for 5 min and then developed for 1 min in a myECL Imager (Thermo). N is represented in figure legend.

### QKI-6 RNA immunoprecipitation

Independent P21 mouse forebrains were dissected in 2 mL of 1X Lysis Buffer (as above). Homogenates were spun at 20,000 × *g* for 20 min and supernatant was collected. 2.5% of the supernatant was saved for western blotting in 1X Laemmli Buffer(Bio-Rad) or RNA extraction. The rest of the lysate was incubated with IgG or QKI-6 coupled streptavidin Dynabeads for 2 h at 4 °C (prepared identically to CLIP protocol). Beads were washed in 1X High Salt Wash Buffer, 4 times for 5 min each with end-over-end rotation. 10% of the beads were resuspended in 1X Laemmli buffer and loaded onto an SDS-PAGE gel to confirm immunoprecipitation of QKI. RNA was extracted from the remaining 90% of beads. For RT-PCR confirmation of QKI-6 targets (Supplementary Fig. 2), primers are available in Supplementary Table 1. Full gel images can be found in the Source Data file.

### Reporting summary

Further information on research design is available in the Nature Research Reporting Summary linked to this article.

## Supplementary information

Supplementary Information

Description of Additional Supplementary Files

Supplementary Data 1

Supplementary Data 2

Supplementary Data 3

Supplementary Data 4

Supplementary Data 5

Reporting Summary

## Data Availability

All raw sequencing data has been deposited on the NCBI Gene Expression Omnibus (GEO) with the following accession numbers: QKI-CLIP data: GSE147119; CRISPR-TRAPSeq data: GSE146935. The mice generated in this study (Strain name: LSL-Rosa26 Cag Cas9 MR04) are available at the Mutant Mouse Resource & Research Center (MMRRC) under the ID: 68058. Source data are provided with this paper.

## Source data

Source Data

## Code availability

Custom code for all analyses within this manuscript is available upon reasonable request. Cell-type-specific analysis^36^ code is publicly available for download at: http://genetics.wustl.edu/jdlab/psi_package/
