# Supplementary material for: Loss of Quaking RNA binding protein disrupts the expression of genes associated with astrocyte maturation in mouse brain
Source: Nat Commun. 2021 Mar 9;12:1537. doi: 10.1038/s41467-021-21703-5 (PMC7943582; doi:10.1038/s41467-021-21703-5)
Supplement: Supplementary file 1 — Supplementary Information [file 41467_2021_21703_MOESM1_ESM.pdf]

SUPPLEMENTARY INFORMATION

Loss of Quaking RNA binding protein disrupts the expression of genes associated with astrocyte maturation in mouse brain.

Supplementary Figure 1

Supplementary Figure 2

Supplementary Figure 3

Supplementary Figure 4

Supplementary Figure 5

Supplementary Figure 6

SUPPLEMENTARY FILES

Supplementary Data 1

Supplementary Data 2

Supplementary Data 3

Supplementary Data 4

Supplementary Data 5

Supplementary Table 1

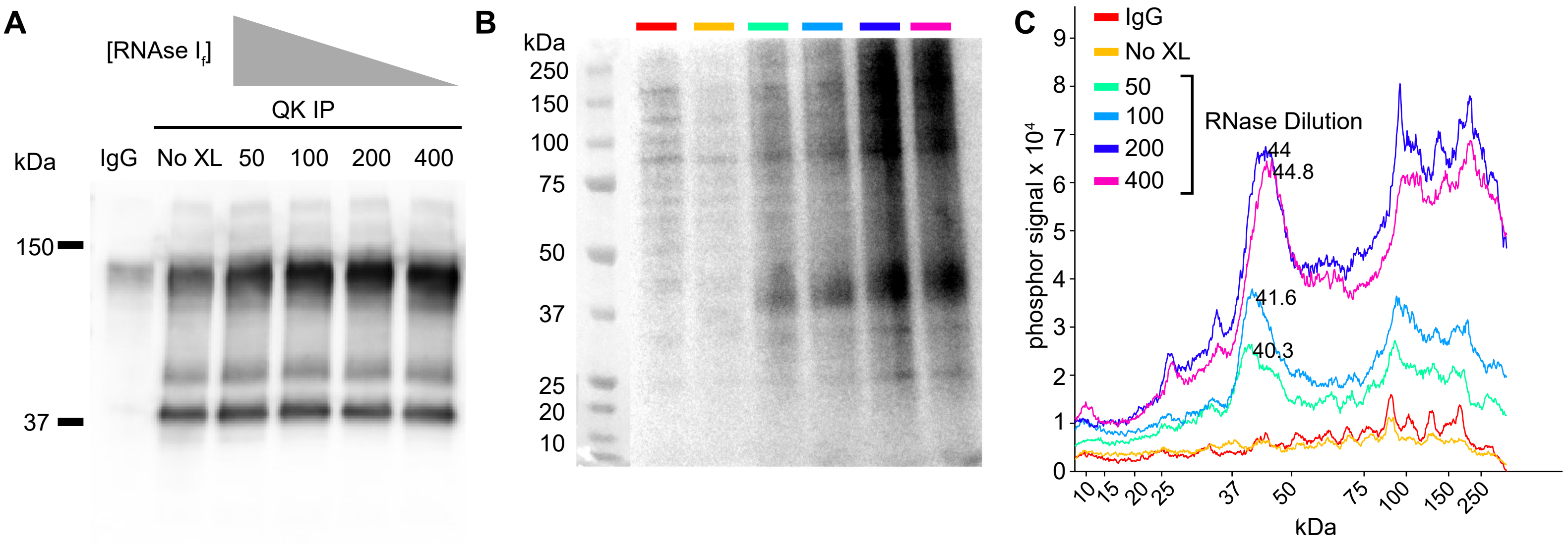

Supplementary Figure 1: Optimization of QKI CLIP protocol. A) Western blot demonstrates that RNAse If digestion does not affect QKI IP. QKI-specific band approx. 38k Da. Band below 150 kDa is IgG heavy and light chains (samples prepared in non-reducing sample buffer). IgG IP does not pull down QK. Values along lanes represent dilution of RNAse If. Experiments were repeated at least 4 times. B) Phosphor screen image of 32P-labeled RNA from QKI IP, across RNAse If dilutions. Colors above lanes correspond to dilutions of RNAse If in plot in panel C. C) Quantification of 32P signal from image in panel B. QKI-bound RNA is around 40-44 kDA, and this area of the gel was excised for sequencing libraries. Source data are provided as a Source Data file.

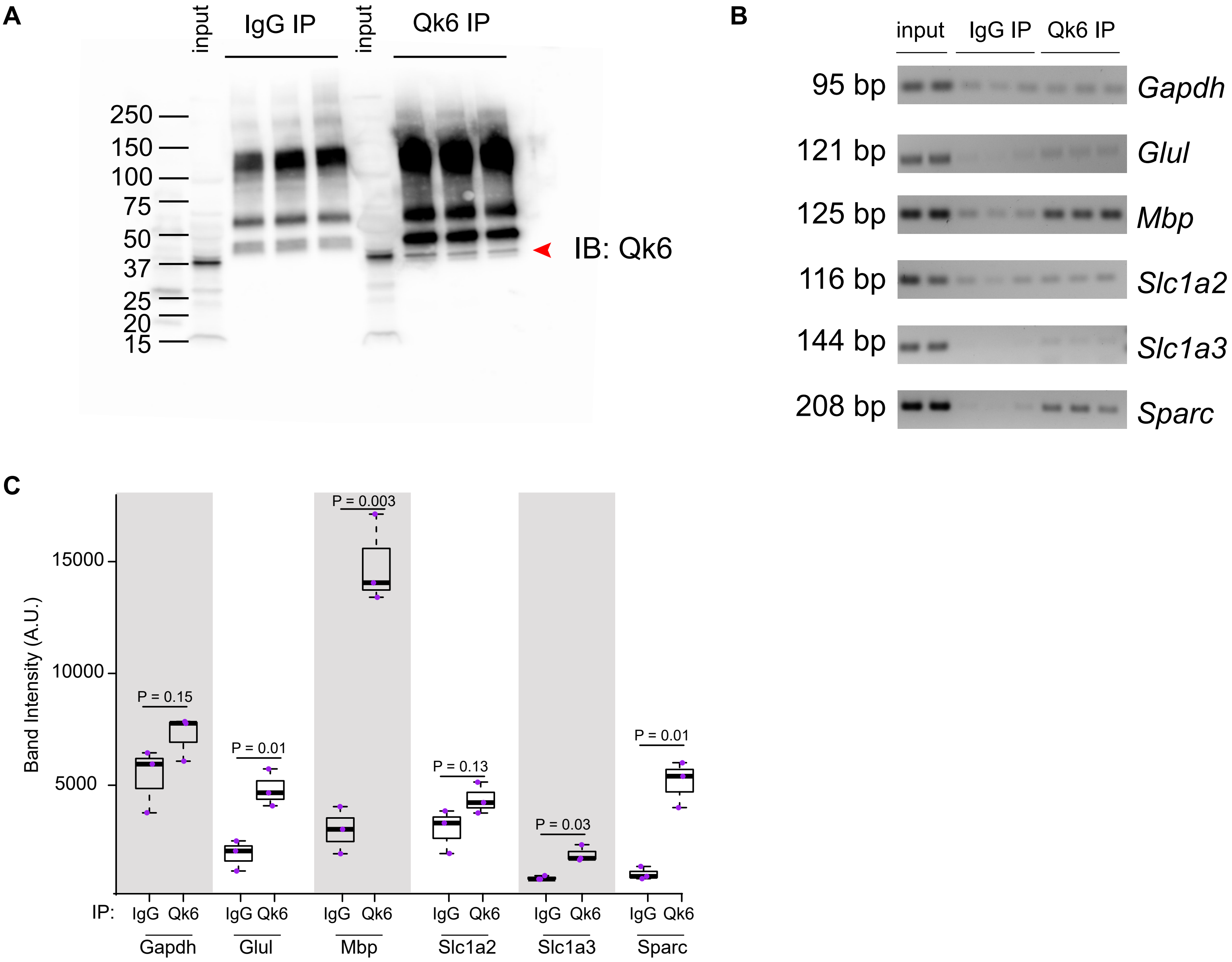

Supplementary Figure 2: Confirmation of CLIP target enrichment. A) Immunoblot of QKI-6 IPs from C57BL/6J mice. IgG IP shows non-specific bands. QKI-6-specific band (38kDa) is seen in the Input samples and in all 3 QKI IPs, indicated by the red arrow, but not in the IgG IPs. Experiments were repeated in at least 3 independent mice. B) RT-PCR gel images. Uncropped gel images are provided in Source Data file. C) Densitometric analysis of RT-PCR gel images. Y axis is arbitrary units. Data are represented by boxplots (center = median, minima and maxima represented by whisker ends, and box bounds representing the 25th and 75th percentiles of the data). Quantification was performed on uncropped, unaltered images. P-values represent two-sided Student's t-tests. Source data are provided as a Source Data file.

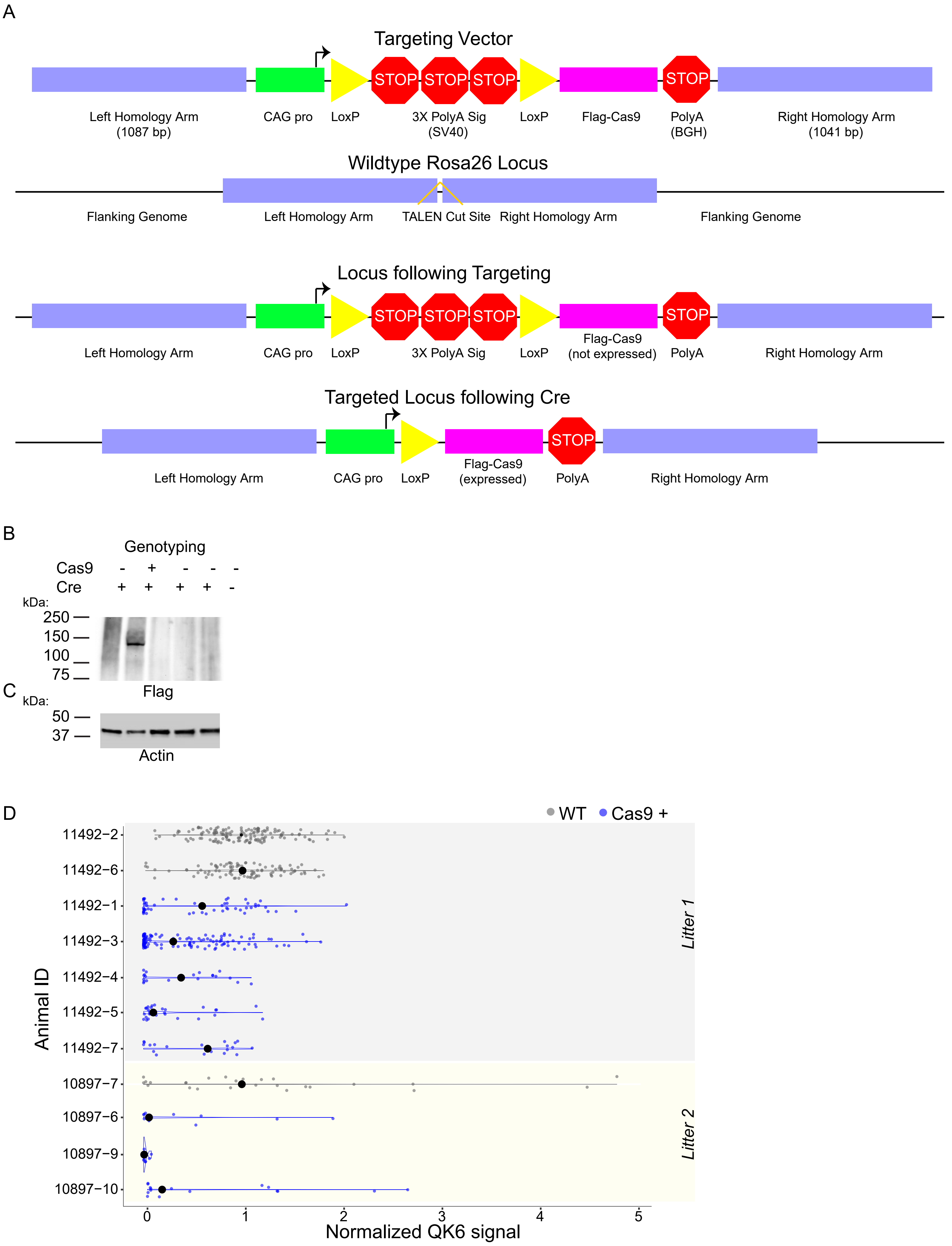
A
Targeting Vector
STOP
STOP
STOP
STOP
Left Homology Arm
(1087 bp)
CAG pro
LoxP
3X PolyA Sig
(SV40)
LoxP
Flag-Cas9
PolyA
(BGH)
Right Homology Arm
(1041 bp)
Wildtype Rosa26 Locus
Flanking Genome
Left Homology Arm
TALEN Cut Site
Right Homology Arm
Flanking Genome
Locus following Targeting
Left Homology Arm
CAG pro
LoxP
3X PolyA Sig
LoxP
Flag-Cas9
(not expressed)
PolyA
Right Homology Arm
Targeted Locus following Cre
Left Homology Arm
CAG pro
LoxP
Flag-Cas9
(expressed)
PolyA
Right Homology Arm
B
Genotyping
Cas9 - + - - -
Cre + + + + -
kDa:
250
150
100
75
Flag
C
kDa:
50
37
Actin
D
WT
Cas9 +
Animal ID
11492−2
11492−6
11492−1
11492−3
11492−4
11492−5
11492−7
10897−7
10897−6
10897−9
10897−10
Litter 1
Litter 2
0
1
2
3
4
5
Normalized QK6 signal

Supplementary Figure 3: Generation of a Cre-dependent Cas9 expressing mouse line. A) Schematic of targeting strategy. TALENS targeting the ROSA26 locus were utilized to insert a targeting vector containing a CAG/Rosa hybrid promoter driving expression of Cas9, interrupted by a Lox-Stop-Lox cassette. Final locus is engineered to express Cas9-Flag in response to Cre recombinase. B) Anti-flag immunoblot detects protein only in brains from mice that genotyped positive for both the Cre allele and the Cas9-Flag Rosa allele. Experiments were performed in > 10 individual founder mice. C) Loading control (anti-actin) showing all lysates contained protein. Experiments were performed in > 10 individual founder mice. Marker indications are in kDa. Images from panels B-C are from the same blot. Uncropped blots for B-C can be found in the Source Data file. D) Quantification of QKI pixels in CFP+ cells across individual animals. Total pixel number per cell was normalized to WT littermates' values, across two litters (labeled by litter 1, grey box and litter 2, yellow box). Median value within genotypes is represented as large black circle. P value represents results of a linear mixed model to account for random effects of individual animals. N = 270 WT, and 280 Cas9+ cells from 3 WT animals and 8 Cas9 animals. Source data are provided as a Source Data file.

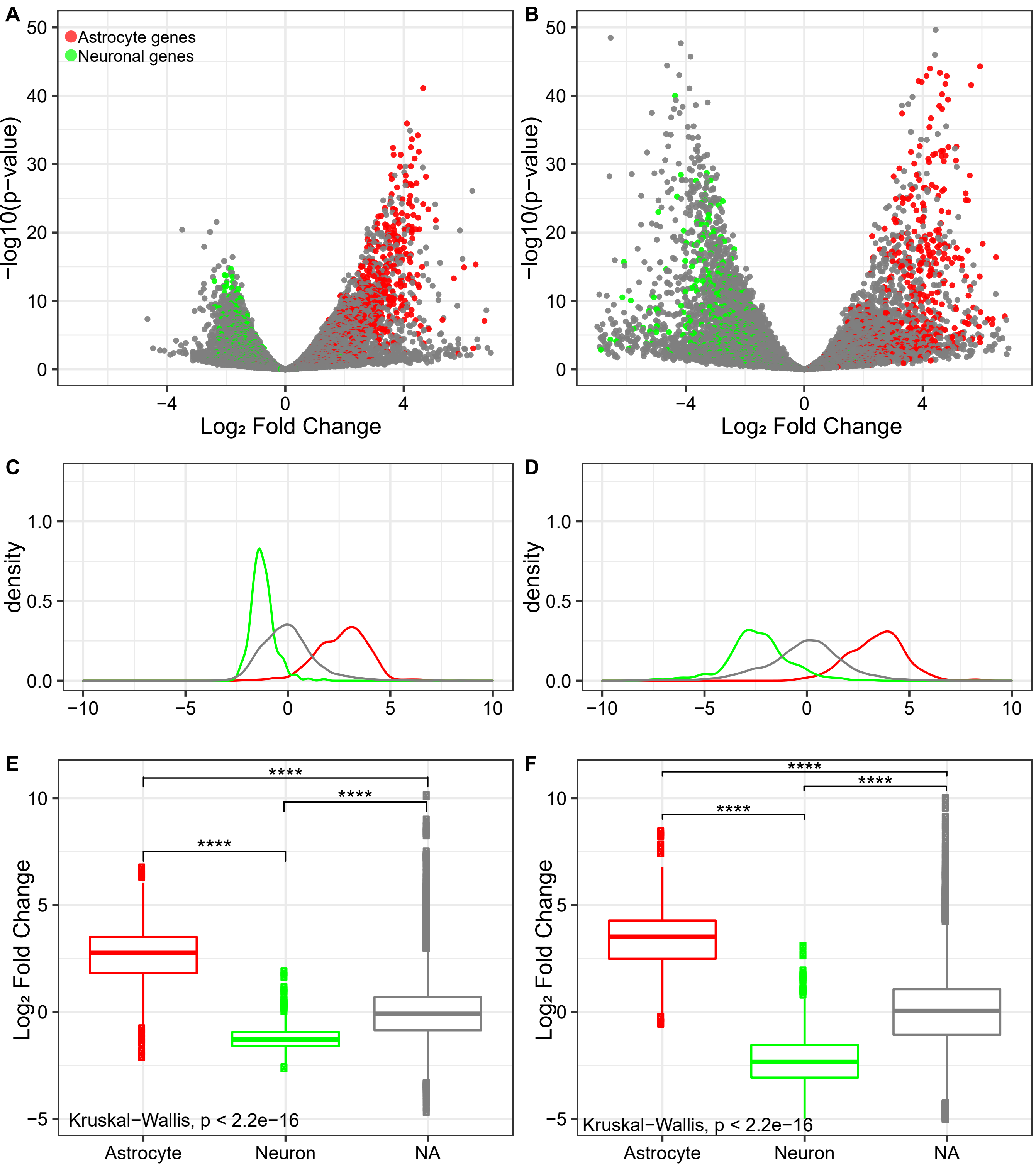
A
B
Astrocyte genes
Neuronal genes
−log10(p−value)
Log₂ Fold Change
C
D
density
E
F
****
Log₂ Fold Change
Kruskal−Wallis, p < 2.2e−16
Astrocyte
Neuron
NA

Supplementary Figure 4: Viral TRAP is comparable to bacTRAP. A,C,E) Viral CRISPR-TRAPseq. B,D,F) Aldh1l1 bacTRAP. A,B) Volcano plots showing enrichment and depletion of known astrocytic (red) and neuronal genes (green). C,D) Density of transcripts representing each enrichment. E,F) Boxplots showing the fold change of known marker genes (astrocytic, neuronal, or other (NA)). N = 580 astrocytic genes, n = 477 neuronal genes, and n = 13,159 other genes. The significance asterisks levels represent Mann-Whitney test. P-values of all comparison are < 2.2e-16.

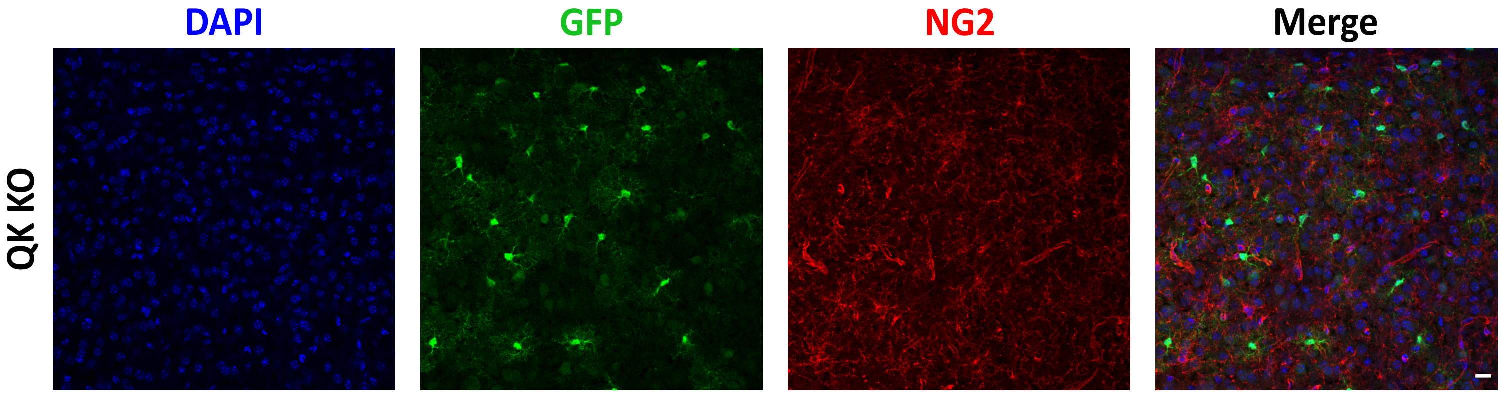

Supplementary Figure 5: QKI mutant cells do not express OPC marker NG2. Immunostaining of GFAP-CFP-Cre positive cells (Green, detected with GFP antibody), in Cas9 + animal does not reveal widespread overlap with Ng2+ OPC cells (red). N=149 CFP+ cells evaluated across images, with only one cell showing an ambiguous Ng2 labeling. Scale bar is 20µm.

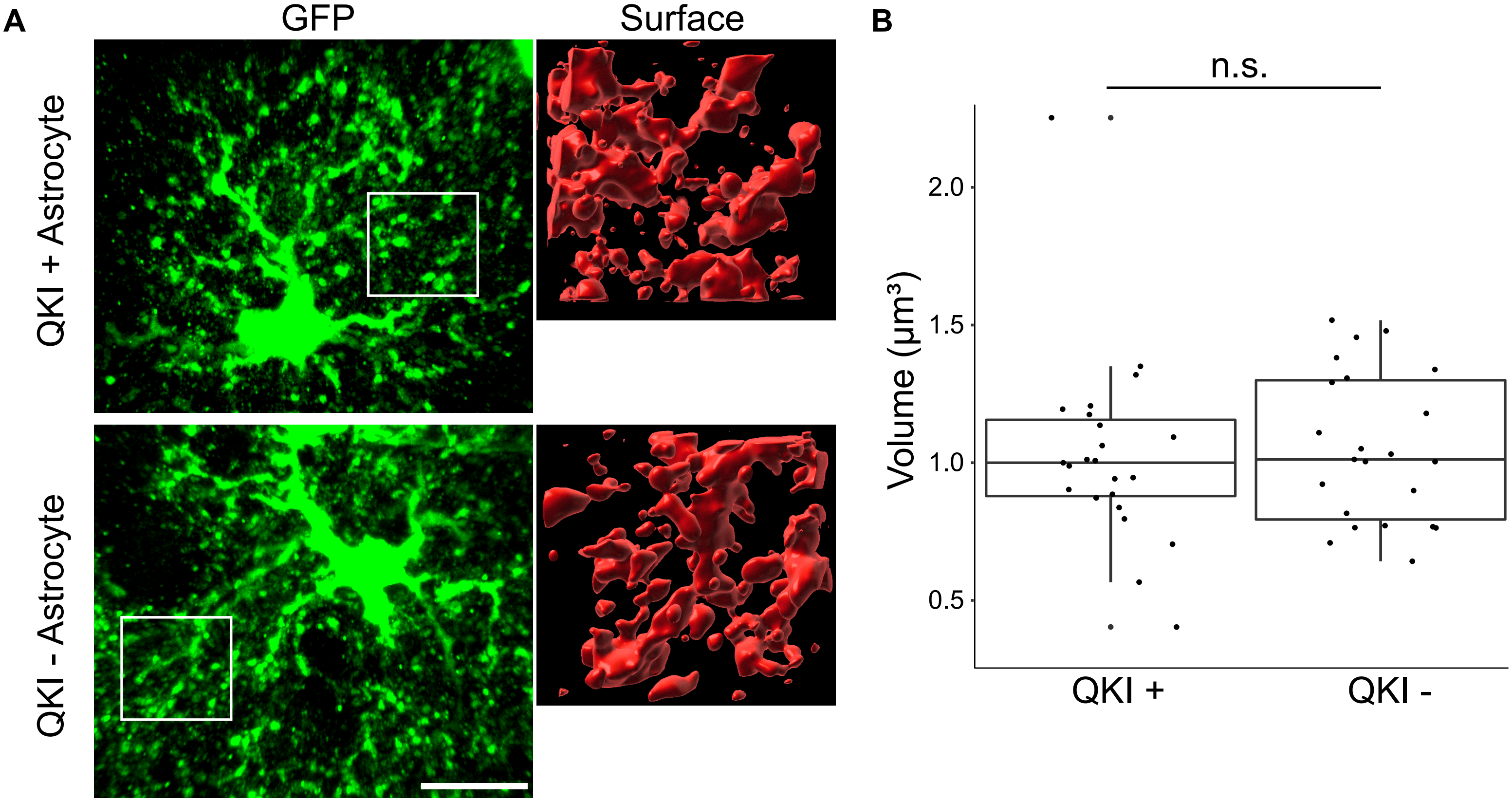

Supplementary Figure 6: QKI KO does not affect astrocyte neuropil infiltration volume. A) Example images of QKI+ and QKI- astrocytes and Imaris surface reconstruction of GFP+ neuropil area. Scale bar is 25μm. B) Quantification of volume surface from each image. Data are represented by boxplots (center = median, minima and maxima represented by whisker ends, and box bounds representing the 25th and 75th percentiles of the data). There is no significant difference between genotypes. Repeated measures ANOVA, $p = 0.59$. Source data are provided as a Source Data file.

Primer Name	Sequence (5' -> 3')
A01m adapter	(/5Phos/rArGrArUrCrGrGrArArGrArGrCrGrUrCrGrUrGrUrArG/3SpC3/)
Rand103tr3 Adapter	(/5Phos/NNNNNNNNNNAGATCGGAAGAGCACACGTCTG/3SpC3/)
Gapdh_RTpcr_For	AGGTCGGTGTGAACGGATTTG
Gapdh_RTpcr_Rev	GGGGTCGTTGATGGCAACA
Glul_RTpcr_For	TGAACAAAGGCATCAAGCAAATG
Glul_RTpcr_Rev	CAGTCCAGGGTACGGGTCTT
Sparc_RTpcr_For	TGGGAGAATTTGAGGACGGTG
Sparc_RTpcr_Rev	GAGTCGAAGGTCTTGTTGTCAT
Mbp_RTpcr_For	TCACAGCGATCCAAGTACCTG
Mbp_RTpcr_Rev	CCCCTGTCACCGCTAAAGAA
Slc1a2_RTpcr_For	TGAATGAAACCATGAACGAGGC
Slc1a2_RTpcr_Rev	GCCGAAAGCAATAAAGAATCCGA
Slc1a3_RTpcr_For	ACCAAAAGCAACGGAGAAGAG
Slc1a3_RTpcr_Rev	GGCATTCCGAAACAGGTAACTC

Supplementary Table 1: Primers used in this study. See Methods for details.
