# Supplementary material for: Loss of Quaking RNA binding protein disrupts the expression of genes associated with astrocyte maturation in mouse brain
Source: Nat Commun. 2021 Mar 9;12:1537. doi: 10.1038/s41467-021-21703-5 (PMC7943582; doi:10.1038/s41467-021-21703-5)
Supplement: Supplementary file 2 — Description of Additional Supplementary Files [file 41467_2021_21703_MOESM2_ESM.pdf]

**Descriptions of Additional Supplementary Files**

**Supplementary Data 1:** QKI-6 bound regions.

Column headers: logFC, logCPM, LR, PValue, & FDR describe results comparing QKI-6 CLIP enrichment over both Input and IgG controls or EdgeR analysis of candidate QKI-6 bound peaks. GeneFeature, utr3match, utr5match, intronmatch, cdsmatch, & intergenic describe the gene annotations overlapping with the peak. Sequences, start, & end describe the sequence of the 50bp peak, as well as coordinates.

**Supplementary Data 2:** GO analysis of CLIP targets

BinGO output on all genes bound by QKI-6, reporting any of the full GO categories with an FDR p<.05. Headers as described by BinGO. Statistical analysis was performed in Cytoscape, using a hypergeometic test with Benjamini-Hochberg correction for multiple comparisions.

**Supplementary Data 3:** Genes with differential ribosomal association following QKI deletion in maturing astrocytes. EdgeR output for all transcripts with p<.05 from likelihood ratio tests (LRT) in this contrast. Headers as above.

**Supplementary Data 4:** Isoform level analysis of differential ribosomal association following QKI deletion in maturing astrocytes.

Limma results for all transcript isoforms with p<.05. Column headers: Ensembl_Transcript_ID and Transcript_Symbol describe the ID of the isoform and the Entrezgene_ID, Ensembl_Gene_ID, Gene Symbol, and Description describe the parent gene of each isoform. Each isoform lists the linear fold-change, log2 fold-change with confidence intervals, p-value, and Benjamini-Hochberg adjusted p-value.

**Supplementary Data 5:** GO analysis on transcripts altered by QKI deletion.

BinGO output(FDR p<.005 cutoff) on all transcripts upregulated following QKI deletion. Headers as described by BinGO. Statistical analysis was performed in Cytoscape, using a hypergeometic test with Benjamini-Hochberg correction for multiple comparisions.
